# Diabetic Retinopathy: Battling the Global Epidemic

**DOI:** 10.1167/iovs.16-21031

**Published:** 2016-12

**Authors:** Arup Das

**Affiliations:** Department of Surgery, Division of Ophthalmology, University of New Mexico School of Medicine, Albuquerque, New Mexico, United States

**Keywords:** diabetic retinopathy, diabetes mellitus, blood–retinal barrier, retina, endothelial cells, pericytes

Blindness is increasing rapidly worldwide due to diabetes mellitus, a devastating disease that has assumed the proportions of a global epidemic. Over 415 million people in the world have diabetes, and it is estimated that this number will reach around 642 million by 2040.^1^ Early detection and prompt treatment are still the gold standards of managing diabetic retinopathy (DR), and landmark clinical trials have shown that such treatment can effectively decrease visual disability by 90%. Despite tremendous progress in the developments of treatment for this disease, many questions remain unanswered.

To further focus on an integrated approach to fight this global epidemic, the ARVO Conference titled “Diabetic Retinopathy: Battling the Global Epidemic” was organized on August 27 and 28, 2015, at the National Institutes of Health (NIH) campus, Bethesda, Maryland. This 2-day conference served as the continuation of the first ARVO Conference on DR (“Diabetic Retinopathy: Approaches to a Global Epidemic”) that was held at the same location in 2010. The 2-day meeting brought together basic and clinician scientists, students, and established investigators from the United States and overseas. The objectives of this meeting were to summarize current concepts about molecular mechanisms and laser and surgical treatments of DR, to discuss current clinical trials of new pharmacotherapies and drug delivery systems, to describe the new concepts on the horizon, and to identify novel targets and formulate research strategies to develop new therapies and clinical trials.

The 2-day conference comprised the following eight sessions: (1) Global epidemic, (2) Molecular mechanisms I, (3) Cellular targets, (4) Imaging and biomarkers, (5) Molecular mechanisms II, (6) Systemic factor control, laser and surgery, (7) Pharmacotherapies, and (8) Future directions. The current review summarizes these new ideas and concepts presented by speakers at the conference, and helps in formulating strategies for fighting this global epidemic. The meeting also included a special lecture, “Regulation of Physiological and Pathological Vascularization by Hypoxia-Inducible Factors,” by Gregg L. Semenza, MD (Johns Hopkins University, Baltimore, MD, USA).

## Global Epidemic

The conference started with an introduction to the magnitude of the global epidemic of diabetes mellitus presented by Lloyd P. Aiello (Joslin Diabetes Center, Boston, MA, USA). Every 9.9 seconds someone in the world develops diabetes. In the United States, approximately 11.3% of adults have diabetes. China has the largest diabetes epidemic in the world, followed by India. One of every three persons with diabetes globally resides in China. The Chinese are developing the metabolic disease at a lower body mass index than the Americans, resulting in an earlier onset of obesity-linked disease. Diabetic retinopathy, a microvascular complication of diabetes, is prevalent in approximately 35% to 49% of diabetics.^2^ Aiello pointed out that Singapore, one of the fastest-growing populations in the Association of Southeast Asian Nations (ASEAN), will become the ninth largest diabetes population by 2030. It is unfortunate that patients with diabetic complications commonly remain unaware of the problem for many years. The lack of patient awareness is considered a major contributing factor for nonadherence to eye care guidelines and poor visual outcomes.^3^ In a recent study with 2853 patients, as expected, only 2% of patients with no retinopathy reported awareness of retinal complications. Surprisingly, 93% of patients with mild retinopathy and 63% of patients with vision-threatening retinopathy were unaware. In patients with a scheduled follow-up with no or mild disease, adherence to recommendations exceeds 90%, but for patients with vision-threatening retinopathy, nearly 71% do not receive timely eye care. The adherence to minimum recommended diabetes eye care means at least an annual ophthalmic examination.

The prevalence of diabetes in India has increased almost 5-fold in the last 30 years, with 13% in the urban and 7% in the rural populations. Tarun Sharma (Sankara Nethralaya, Chennai, India) cautioned that Indians have the highest incidence rate of diabetes, with rapid conversion from normoglycemia to dysglycemia by 45% as shown in the CURES Study (Chennai Urban Rural Epidemiologic Study).^4^ The Sankara Nethralaya Diabetic Retinopathy Epidemiology and Molecular Genetics Study (SN-DREAMS-I), a cross-sectional study, reported the prevalence of obesity indices in the urban south Indian population. Isolated abdominal obesity and higher WHR (waist-to-hip ratio) in women were associated with DR, but not with the severity of DR.^5^ Sharma stressed that the Asian diabetes phenotype (the largest number of diabetics in the 40- to 59-year age group) is distinctly different from the European diabetes phenotype (the largest number of diabetics in the >60-year age group). Despite low body mass index, Asians are more likely than their Caucasian counterparts to develop diabetes mellitus; this is partly due to an increased propensity to store fat viscerally rather than subcutaneously. Asians are also more insulin resistant than non-Asians, with increased concentration of free fatty acids and inflammatory markers, and have lower beta cell function to overcome insulin resistance than non-Asians.

## Molecular Mechanisms

### Epigenetics

Clinical and experimental studies have clearly documented that hyperglycemia is the major instigator in the development of diabetic complications, implying that the lowering of glycated hemoglobin (HbA1c) level should attenuate microvascular complications. However, recent analysis of data from the Diabetes Control and Complication Trial (DCCT) by the Epidemiology of Diabetes Interventions and Complications (EDIC) Research Group has shown that the mean HbA1c explains less than 11% of the risk of retinopathy, implying that 89% of the variation in risk could be due to hyperglycemia-mediated factors that the mean HbA1c value fails to capture.^6^ Michael Brownlee (Albert Einstein Medical College, New York, NY, USA) discussed the novel hypothesis that the major determinant of complications not captured by HbA1c could be due to spikes in hyperglycemia. These spikes are high enough to activate persistent oxidant production during subsequent normal glycemic phases, but are generally too brief to alter HbA1c levels.^7^ He discussed interesting results showing that activation of a multicomponent feedback loop, via mitochondrial superoxide-induced release of free iron and H_2_O_2_, activates protein phosphatase 2 and dephosphorylates Akt1. Reduced Akt1 activity decreases inhibitory phosphorylation of glycogen synthase kinase (GSK)-3β, resulting in increased phosphorylation of voltage-dependent anion channel (VDAC). The results also showed that the transient exposure of cells to high glucose (6 hours) shifts the glucose concentration–reactive oxygen species (ROS) curve to the left, inducing persistent mitochondrial superoxide production.

Further discussion focused on the role of epigenetics in the metabolic memory phenomenon. Due to recruitment of methyl-writing and methyl-erasing histone enzymes at the promoter of nuclear transcriptional factor B in high-glucose conditions, while methylation of histone 3 lysine 4 is increased, that of histone 3 lysine 9 is decreased. But when the cell is removed from its hyperglycemic environment, these spikes recruit histone demethylase 1 at the histone 3 lysine 9 of the p65 subunit of the nuclear transcriptional factor B proximal promoter, and disruption of this multicomponent feedback loop by regulating these components reverses the persistent left shift caused by transient hyperglycemia, and normalizes persistent posthyperglycemic increase in nuclear transcriptional factor B promoter methylation at histone 3 lysine 4. Thus, based on the results, Brownlee suggested an urgent need for the development of new biomarkers to complement HbA1c and novel therapeutic agents for the prevention and treatment of diabetes complications.

The diabetic environment increases oxidative stress, which is considered to play a pivotal role in the development of diabetic complications. Reactive oxygen species are elevated in the retinal mitochondria in diabetes, and are shown to initiate many molecular events that are implicated in the development of diabetic complications including retinopathy.^8^ In addition to increased superoxide production, mitochondrial biogenesis is impaired and copy numbers are decreased. Due to lack of supporting histones and the close proximity to superoxide-generating electron transport chain, mitochondrial DNA (mtDNA) is prone to oxidative damage. This mtDNA damage is further exacerbated as this double-stranded DNA also lacks supporting histones. Although mtDNA is very small in size (16.7 kb) and encodes 37 genes, 13 of these genes make proteins essential in the electron transport chain system for oxidative phosphorylation. Due to damage of mtDNA in diabetes, the transcription of mtDNA-encoded genes becomes subnormal, and the futile cycle of superoxide accumulation continues to self-perpetuate.

**Figure 1:**
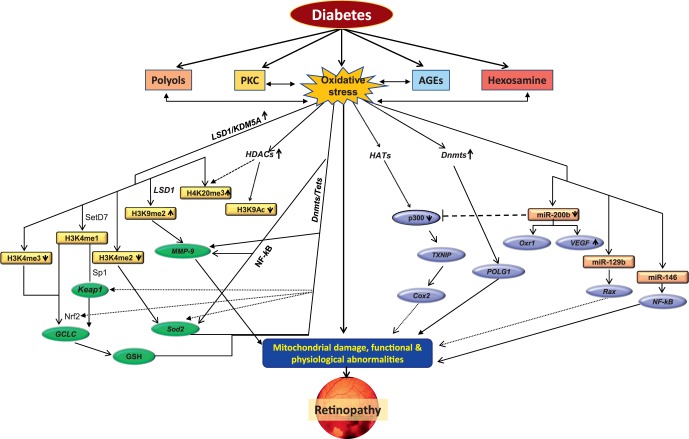
Metabolic abnormalities and epigenetic modifications in diabetic retinopathy. Circulating high glucose increases oxidative stress and alters many metabolic pathways, including increased production of polyols and advanced glycation end products (AGEs), and activates protein kinase C (PKC) and hexosamine pathways. These metabolic pathways, in addition to increasing oxidative stress, are also activated by oxidative stress. Increased oxidative stress damages mitochondria, and damaged mitochondria themselves further fuel into oxidative stress. Enzymes responsible for epigenetic modifications, including for maintaining histone acetylation (HATs and HDACs) and methylation (SetD7 and LSD1) and DNA methylation/hydroxyl-methylation (Dnmts/Tets), are altered, affecting histone modifications and/or DNA methylation in the promoter regions of many genes associated with the development of diabetic retinopathy. Alteration in the transcription of the genes associated with oxidative stress, including Sod2, GCLC, MMP-9, and POLG (gene encoding mitochondrial superoxide dismutase, glutamyl cysteine synthase, matrix metalloproteinase-9, and polymerase gamma for mtDNA biogenesis, respectively), further compromises mitochondrial homeostasis. In addition, many microRNAs (e.g., miR 200b, miR 129b, and miR 146) are also altered, fueling into mitochondrial damage. Damaged mitochondria accelerate capillary cell apoptosis, resulting in cell loss and the development of diabetic retinopathy. By courtesy of Renu Kowluru, PhD.

Because the external factors, including environment and disease state, can also regulate the transcription of a gene without altering the DNA sequence, these epigenetic changes are now appreciated in the development of DR and other diabetic complications. Methylation of cytosine forms 5-methylcytosine, and this process is commonly associated with gene silencing and is facilitated by DNA methyltransferases, a group of redox-sensitive enzymes. Although mtDNA represents less than 1% of the total cellular DNA, it contains 440 CpG sites and 4500 cytosines at non-CpG sites, making it a good candidate for cytosine methylation. Renu Kowluru (Wayne State University, Detroit, MI, USA) discussed the role of mitochondrial epigenetics in the pathogenesis of DR.^9,10^ Data were presented showing increased methylation of retinal mtDNA in diabetes, and this hypermethylation appears to be assisted by impaired translocation of the DNA methylating enzyme inside the mitochondria. The regulatory region of mtDNA, which contains transcription and replication elements, the D-loop region, appears to have a higher degree of hypermethylation than other regions of the mtDNA. Due to increased mtDNA methylation, the transcription of the mtDNA-encoded genes that are important in the maintenance of electron transport chain is compromised, and electrons begin to leak out, further fueling into the vicious cycle of superoxide radicals. Regulation of DNA methylating enzyme ameliorates hyperglycemia-induced decrease in mtDNA transcription and increase in apoptosis. The discussion clearly implicated a critical role of mitochondrial epigenetics in the development of DR, and suggested the possible use of modulators of DNA methylating enzymes in the development/progression of DR (Fig. 1).

### Micro RNAs

Micro RNAs (miRs) represent small noncoding RNAs involved in gene regulation by gene suppression and are involved in physiological and pathologic processes. They also are secreted in exosomes that are released in biological fluids. Micro RNA expression profiles can serve as biomarkers of disease in the vitreous and serum, and can serve as therapeutic targets in the retina. There are several key studies that provide support for the role of specific miRs in DR pathogenesis and that show differential expression in the diabetic retina.^11–14^ Manuela Bartoli (Georgia Regents University, Augusta, GA, USA) discussed the significance of these key miRs in DR. Diabetic retinopathy is associated with decreased expression of miR 126, miR 146, and miR 200b, which can result in an increase in VEGF expression. Decreased miR 146 can also increase fibronectin that contributes to basement membrane thickening seen in DR. Increased levels of miR 195 reduce silent mating type information regulation 2 homolog (sirtuin 1; Sirt-1). Sirt-1 mRNA is decreased in the diabetic retina compared to age-matched controls. Bartoli explained the concept that diabetes represents accelerated vascular aging. There are certain miRs that are associated with stress-induced premature senescence (SIPS) including mi R21 and mi R34a. Micro RNA 21 expression has been shown to be transcriptionally regulated by signal transducer and activator of transcription (STAT3) and to regulate such processes as angiogenesis and inflammation. Micro RNA 21 can also decrease the expression of the key protease inhibitor, tissue inhibitor of metallprotease (TIMP3). Micro RNA 34a transcriptionally regulates p53 and promotes apoptosis and senescence, inflammation, and vascular dysfunction. Micro RNA 34 is upregulated in diabetes and promotes mitochondrial dysfunction, senescence, and inflammation by downregulating Sirt-1, superoxide dismutase (SOD2), and thioredoxin reductase (TrxR2) in human retinal endothelial cells. Bartoli's talk raised the following questions: Can we translate experimental tools for drugging miRNAs to the clinic? What will that approach look like—adneo-associated virus (AAV) or anti-miR oligonucleotides?

### Inflammation

Elevated inflammatory mediators such as VEGF, IL-8, IL-1β, IL-6, and TNFα are observed in experimental models of diabetes^15–17^ and are elevated in vitreous of diabetic individuals.^18–20^ Importantly, inhibition of these inflammatory mediators slows progression of retinopathy in experimental models of diabetes.^21,22^ John Penn (Vanderbilt University, Nashville, TN, USA) presented the novel effects of arachidonic acid metabolites, the proinflammatory prostaglandins and thromboxanes, and the anti-inflammatory, epoxyeicosatrienoic acid (EET) and epoxydocosapentaenoic acids (EDPs). The anti-inflammatory EETs have been shown to be reduced in the vitreous of diabetic individuals.^23^ The anti-inflammatory 11,12-EET and 19,20-EDP inhibit palmitic acid–induced inflammation in Müller cells. Key enzymes in the generation of these anti-inflammatory EETs are CYP2C and CYP2J and the soluble epoxide hydrolase (sEH). High glucose level decreases the expression of CYP2C and CYP2J, and inhibition of these enzymes in Müller cells results in the release of key proinflammatory factors including TNFα, IL-1β, IL-6, and IL-8. Agents such as GSK2265964, a sEH inhibitor, inhibited palmitic acid–induced inflammation in Müller cells. 11,12-EET inhibits nuclear factor-kappa B (NF-κB) translocation in endothelial cells. Both EET and EDP inhibit TNFα-induced vascular cell adhesion molecule (VCAM)-1, intercellular adhesion molecule (ICAM)-1, and E-selectin expression and leukocyte adherence both in vitro and in vivo. These findings indicate that the lipidomic fingerprint of diabetic patients might be a clinical biomarker of DR.

Apart from VEGF, many other cytokines are shown to be involved in the pathogenesis of DR. The angiopoietin-Tie pathway is one of those other key pathways in which angiopoietin-2 acts as a proangiogenic factor and angiopoietin -1 (Ang-1) as an endogenous inhibitor. Bala Ambati (University of Utah, Salt Lake City, UT, USA) discussed the use of a single intravitreal dose of adeno-associated virus serotype 2 encoding a stable and potent form of angiopoietin-1 (AAV2.COMP-Ang1). It can ameliorate several key histologic features of DR in Ins2Akita mice. In early DR, AAV2.COMP-Ang1 corrected leukocyte–endothelial interactions, retinal oxygenation, vascular density, vascular marker expression, vessel permeability, retinal thickness, and inner retinal cellularity. In late DR, AAV2.COMP-Ang1 enhanced the therapeutic benefit of intravitreally delivered endothelial colony-forming cells, a key vascular-derived progenitor population, by promoting their integration into the vasculature.^24^ This puts forth the idea of gene therapy for DR and that AAV2.COMP-Ang1 single dose could be considered in the future as a targeted therapy to prevent neurovascular pathology, support vascular regeneration, and even stabilize vision in DR.

### Neuroprotection

Elia Duh (Johns Hopkins University, Baltimore, MD, USA) considered the key endpoints of DR including oxidative stress, increased inflammatory cytokines, blood–retinal barrier dysfunction, and neural degeneration. Photoreceptors are an important source of diabetes-induced ROS and contribute to the proinflammatory retinal environment in DR.^25^ Neurons contribute to increasing permeability in DR.^26^ Duh emphasized that promoting protective factors in the eye is a viable therapeutic strategy for DR and neurodegeneration. He discussed the nuclear factor erythroid 2–related factor (Nrf2), a transcription factor that binds to the antioxidant response element (ARE) to activate transcription of multiple genes. Nrf2 is bound to Keap1 and is tethered to actin cytoskeleton in the cytoplasm. Oxidative stimuli interact with Keap1 (modifying cysteine residues) and release Nrf2 and allow it to translocate to the nucleus.^27^ Nrf2 is activated early in the retina in streptozotocin (STZ)-induced diabetes (8 weeks) with an increase in nuclear translocation of Nrf2. Nrf2 loss of function results in an earlier onset of DR and an exacerbation of multiple retinal endpoints.^28,29^ Nrf2 loss of function significantly exacerbates neurodegeneration in the retinal ischemia–reperfusion model.^29^ Because Nrf2 is in activated in DR, the Duh group pursued strategies to enhance or activate Nrf2 to slow progression of DR.^30–32^ Nrf2 is negatively regulated by Keap1, and activation of Nrf2 can occur by drugs that modulate Keap1 including triterpenoids (CDDO-Im, CDDO-Me), fumaric acid esters (dimethyl fumarate/DMF Tecfidera), and the biologically and pharmacologically active metabolite, momomethyl fumarate (MMF). Evaluation of these agents in the retinal injury models^29^ shows that MMF improves neuronal function (ERG) in this model in a Nrf2-dependent fashion. Duh's work highlights the importance of neurodegeneration as an early event in DR and that neuroprotection is a possible strategy for treatment of DR. Nrf2 is an endogenous protective mechanism for DR, and pharmacologic activation of Nrf2 may represent an attractive therapeutic strategy for DR.

### Endogenous Protective Factors

Poor glycemic control and poor blood pressure and lipid control are known systemic factors in development and progression of DR. However, the 50-year Joslin Medalist Study that included type 1 diabetics with longer than 50 years of diabetes duration showed that approximately 40% of them are protected from diabetes complications (no retinopathy or mild retinopathy) and allow identification of endogenous protective factors.^33^ George King (Joslin Diabetes Center, Boston, MA, USA) discussed “protective factors” found in this population. Increased risk of proliferative diabetic retinopathy (PDR) was seen in subjects with high carboxy-ethyl (CEL) and pentosidine as compared with the rest of the cohort. Importantly there appeared to be different protective factors for the retina and kidney.

## Cellular Targets

### Blood–Retinal Barrier

**Figure 2:**
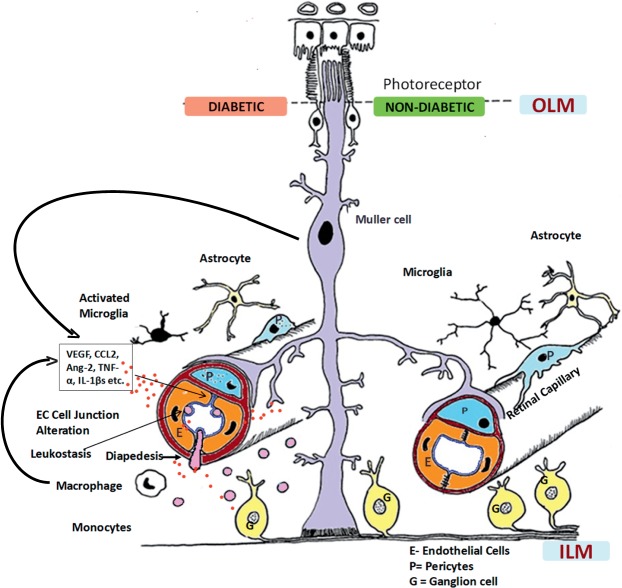
Neurovascular unit of the retina in nondiabetic and diabetic conditions. Normally, pericytes and endothelial cells constitute the blood–retinal barrier (BRB) (retinal capillary) that is covered intimately by multiple processes of the Müller cells (*right*). Astrocytes and microglia with long processes also surround the capillaries, maintaining the normal homeostasis for neuronal signaling and synaptic transmission. In diabetes, chronic inflammation sets in, contributing to the breakdown of the BRB (*left*). Müller cells and endothelial cells produce chemokines (including monocyte chemoattractant protein-1) that lead to increased leukostasis, diapedesis, and influx of monocytes into the retina and increased production of cytokines, including vascular endothelial growth factor (VEGF), tumor necrosis factor (TNF)α, interleukin (IL)-1β, matrix metalloproteinase, and angiopoietin (Ang)-2. These inflammatory mediators then result in the breakdown of endothelial cell–cell junctions. Microglia become activated, and there is increased apoptosis of ganglion cells and amacrine cells, which deranges synaptic degeneration. In retinal capillaries, pericyte dropout and thickening of the basement membrane also occur as a result of hyperglycemia, all contributing to increased leakage from vessels. Photoreceptors contribute to production of superoxide and inflammatory proteins in this process. CCL-2, chemokine ligand 2; EC, endothelial cell; ILM, inner limiting membrane; OLM, outer limiting membrane. Reprinted with permission from Das A, McGuire PG, Rangasamy S. Diabetic Macular Edema: Pathophysiology and Novel Therapeutic Targets. *Ophthalmology*. 2015;122:1375–1394. Copyright © 2015 American Academy of Ophthalmology. Published by Elsevier, Inc. All rights reserved.

The retina has a complex structure with four major cell types: vascular (pericytes and endothelial cells), macroglial cells (Müller cells and astrocytes), neurons (photoreceptors, bipolar cells, amacrine and ganglion cells), and microglia (which act as phagocytes). Its vasculature is separated from the surrounding neural components by cytoplasm of Müller cells and glial cells. The capillaries are surrounded by a connective tissue sheath, which is mainly composed of collagen types IV and V, laminin, and heparan sulfate proteoglycan core protein.^34^ The inside of the basement membrane is lined by a single layer of endothelial cells, and the outside is supported by pericytes.^35^ Membranes between adjacent endothelial cells fuse to form the tight junctional complexes, and these junctions form the blood–retinal barrier, which is crucial for the regulation of the retinal microenvironment. These tight junctions consist of proteins that help in the organization and structure; changes in these complexes in diabetes result in blood–retinal barrier breakdown altering the vascular permeability and macular edema (Fig. 2).

### Cell–Cell Junctions

Proper vision is maintained by a well-functioning tight junction system as it impedes diffusion of macromolecules from blood to the retina. Occludins are associated with the endothelial cell barrier properties, and zonula occludins are essential in retinal junctional organization. Due to breakdown of blood–retinal barrier in diabetes, blood vessels become leaky. David Antonetti (University of Michigan, Ann Arbor, MI, USA) discussed alterations of junctional complexes in the diabetic environment.^36^ Using an ischemia–reperfusion rat model, his results suggested that although zonula occludin-1 protein content is not decreased, its distribution at the blood–retinal barrier is altered in diabetes, and this alteration in zonula occludin-1 localization is accompanied by a rapid phosphorylation of occludin at Ser490, which coincides with the altered organization and increased vascular permeability.

The presentation clearly highlighted the importance of VEGF in ischemia–reperfusion–induced permeability and phosphorylation of occludin, as administration of bevacizumab prevented ischemia–reperfusion injury–mediated vascular permeability. The process appears to be mediated via preventing the phosphorylation of occludin at Ser490 and vascular endothelial growth factor receptor (VEGFR)-2 phosphorylation at Tyr1175. The discussion about the retinopathy timeline in ischemia–reperfusion suggested that although permeability and VEGF-receptor activation occur within a few minutes of ischemia–reperfusion, phosphorylation of occludin takes longer, and distribution of zonula occludin-1 at the blood–retinal barrier follows occludin phosphorylation. The barrier becomes dysfunctional, and within hours of ischemia–reperfusion the inflammatory genes begin to increase and leukocyte infiltration is initiated. As an atypical protein kinase C signaling is involved in the VEGF-induced endothelial permeability and its inhibitors block proinflammatory cytokine-induced vascular permeability, the novel data also showed that the same inhibitor also prevents ischemia–reperfusion–induced vascular permeability.

Further discussion included the role of Norrin, a TGF-β family member that is produced by glia and functions as a high-affinity Wnt-like ligand for its endothelial cell receptor Frizzled4, in the blood–retinal barrier.^37^ Norrin rescues VEGF-induced permeability in retinal endothelial cells, and this is mediated via induction of Norrin receptors and coreceptors by Norrin. Also, absence of canonical Wnt signaling arrests endothelial cell invasion near the neuroepithelial surface and produces clusters of endothelial cells (glomeruloid bodies), but not vascular plexus, suggesting that canonical pathway is required for barrier function, and GSK3 inhibition may not be sufficient to restore ion barrier and solute barrier.

### Pericytes

Endothelial cells and pericytes are the major cellular components of the retinal capillaries, and in normal retinal capillaries, their ratio is 1:1. This ratio of endothelial cells to pericytes is highly controlled by a series of signaling pathways operating in an autocrine and/or paracrine manner; and several molecules, such as platelet-derived growth factor, TGFβ, VEGF, and angiopoietins, are involved in modulating the interactions between pericytes and endothelial cells. In diabetes, pericytes begin to drop off, and the ratio falls down to 4:1, and due to loss of pericytes, capillaries become nonperfused. Patricia D'Amore (Harvard University, Boston, MA, USA) discussed the role of pericytes in the development of background DR, and presented her recent work using nondiabetic mice conditionally expressing a diphtheria toxin receptor in mural cells. Results from this model strongly suggested that the loss of pericyte is spatially and temporally associated with the development of histopathologic lesions characteristic of retinopathy, including formation of microaneurysms, degenerative capillaries, and increased vascular permeability.^38^ Since formation of acellular capillaries results in capillary nonperfusion, due to ischemia, VEGF expression is increased, resulting in neovascularization. The loss of pericytes removes/attenuates the inhibitory effect that the pericytes have on endothelial migration and proliferation, and this makes the retinal vasculature more vulnerable to VEGF stimulation. Her work concluded that pericyte loss, an early event in the pathogenesis of DR, could be sufficient to destabilize the vasculature, which would increase vascular permeability and contribute to the histopathologic lesions of DR.

### Basement Membrane

The basement membrane, a highly organized structure, has multiple components; while fibronectin, a large glycoprotein, functions as a molecular glue to keep the components organized, the binding sites on type IV collagen directly interact with integrins, laminins regulate cellular function, and heparan sulfate proteoglycan interacts with other components of the basement membrane to mediate cell attachment. Basement membrane thickening is seen before other histopathology associated with DR can be observed, and is considered one of the hallmarks of diabetic microvascular lesions. This thickening appears to be due to increased synthesis/decreased degradation of its major components. Sayon Roy (Boston University, Boston, MA, USA) showed that increased production of fibronectin and collagen IV in high glucose induces apoptosis and facilitates permeability of solutes, inducing apoptosis.^39^ Regulation of endothelial cell membrane by antisense oligos in rodents reduces basement membrane thickening and vascular permeability in the retina, and it also decreases the formation of degenerative capillaries pericyte ghosts.

To understand the causal relationship between basement membrane thickening and increased permeability in diabetes, Roy discussed the role of an extracellular enzyme, lysyl oxidase, in increased basement membrane permeability.^40^ This enzyme catalyzes formation of aldehydes from lysine residues in collagen and elastin precursors, and its increased enzyme expression and activity are considered to play a role in increased invasiveness of tumor cells, possibly, in part, by mediating its effects on the structure and physical properties of the endothelial cell membrane. The results were presented showing a close association between increased lysyl oxidase and vascular permeability in retinal endothelial cells in high-glucose conditions, which could be ameliorated by specific small (or short) interfering RNA (siRNA) of lysyl oxidase. Overall, this presentation had a clear message that the integrity of the basement membrane appears to require an optimal degree of lysyl oxidase–dependent cross-linking, and the basement membrane thickening could serve as a potential therapeutic target to prevent vascular leakage and lesions characteristic of DR.

### Photoreceptors

Although DR had long been considered as a microvascular disease, recent studies have suggested that retinal photoreceptors, the most prevalent cells in the retina, also play a major role in its development. The importance of photoreceptors in DR is supported by some previous observations showing that the density of retinal vasculature in diabetic animals lacking photoreceptors is partially preserved, and retinitis pigmentosa patients present less DR.^41^ Timothy Kern (Case Western University, Cleveland, OH, USA) discussed the importance of photoreceptors in the development of DR. As photoreceptors are the major consumers of oxygen in the retina, and inhibition of oxidative stress ameliorates the vascular lesions of early DR, Kern discussed how the generation of retinal superoxide is significantly higher in dark, which is further exacerbated in diabetes. In diabetes, photoreceptors are one of the major sources of increased ROS, and these oxidants appear be generated by both mitochondrial and cytosolic sources. Mice genetically manipulated for opsin are protected from diabetes-induced increase in ROS and increase in proinflammatory molecules in the retina, suggesting an important role in the development of DR.^25^ Mutation of proline on position 23 in rhodopsin induces rhodopsin misfolding and photoreceptor degeneration; the data showed that diabetic mice with this proline mutation substituted with histidine (P23H) and are therefore protected from retinal capillary degeneration, further strengthening the role of photoreceptors in DR.

Photoreceptors and RPE are critical components of the visual cycle and both act as a single unit in the visual cycle; the effect of administration of RPE65 inhibitor, retinylamide, on the formation of degenerative capillaries in the retina was also discussed.^42^ Administration of retinylamide, in addition to inhibiting diabetes-induced increase in albumin extravasation into the nonvascular retina, also inhibits capillary degeneration and attenuates the accumulation of superoxide and expression of inflammatory proteins. Although retina is an extension of the brain and is formed embryonically from neural tissue, its vasculature escapes lesions in diabetes, and the discussion further focused on the role of photoreceptors in the retinal vascular lesions. Kern showed that treatment with light in the far-red to near-infrared region of the spectrum decreases oxidative stress and ameliorates lesions of DR, and work from others shows the reversal of diabetic macular edema by low-intensity green light all night to suppress dark current in photoreceptors. The present study strongly implied an important role of photoreceptors in the development of DR and suggested that the light could be used to influence its development.

### Microglia

Further addressing the role of nonvascular cells in DR, Susanne Mohr (Michigan State University, Lansing, MI, USA) discussed the role of microglia, the resident immune cells in the retina. These cells are responsible for immune surveillance and maintaining the microenvironment in general; however, through their contact with other retinal cells including neurons and glia cells, microglia recognize damaged cells to regulate repair processes and remove debris via phagocytosis. Although functions of microglia overlap with those of other glia cell types, these cells with mesodermal origin are fundamentally different astrocytes and Müller cells, and resemble more monocytes, or more precisely, the resident macrophages. As mentioned above, DR is now also appreciated as a low-grade chronic inflammatory disease; the presentation focused on the role of microglia in regulation of chronic inflammatory events leading to the potential breakdown of the vasculature and retinal cell death. The discussion included her work showing that proliferation and migration of microglia correlate with events associated with chronic inflammation, including production of cytokines and chemokines, increase in ROS, and cell death. As a functional CX3CR1 (receptor on microglia)/CX3CL1 (ligand presented by neurons) axis is crucial for healthy crosstalk between microglia and neurons, and this receptor/ligand binding suppresses production of inflammatory mediators by microglia, Mohr presented results from her latest studies focused on understanding the role of CX3CR1/CX3CL1 axis in the development of DR. Disruption of this axis by changes in the CX3CR1 receptor, or cleavage of the CX3CL1 ligand, results in neuroinflammation and subsequent neurodegenerative diseases. CX3CR1 knockout on microglia in mice has greater microglia activation compared to that in wild-type diabetic mice and also increased the number of acellular capillaries, a hallmark of histopathology associated with DR.^43^ Although Mohr's data highlighted the importance of focusing on microglia behavior in DR, due to limited knowledge/literature about the role of microglia in retinopathy, several important questions remain, including how many types of microglia are present in the healthy and diabetic retina. Is this activation always a bad event, or is this dependent on the microenvironment? How does crosstalk with other glia cells influence microglia behavior, and what is the effect of diabetes-associated systemic changes on microglia function?

## Imaging and Biomarkers

Currently, optical coherence tomography (OCT) is used routinely as a guide for treatment of diabetic macular edema (DME) for quantitative measurement and mapping of macular thickening. S. Burns (Indiana University, Indianapolis, IN, USA) described the adaptive optics scanning laser ophthalmoscopy (AOSLO) imaging of the human retina as a futuristic tool for management of DR. The AOSLO imaging corrects optical imperfections and allows us to image the retinal cells. Blood vessels and the blood within can scatter light strongly, and a type of dark-field imaging can accentuate index of refraction borders to improve vascular imaging.^44^ Even in early diabetes, capillary dropout and changes in capillary diameter (both dilation and constriction) occur. Burns showed examples of remodeled capillaries, microaneurysms, low capillary density, capillary dropout, blood flow stasis, capillary engorgement, and tortuosity in mild and moderate nonproliferative DR using the AO imaging.^45,46^ Other retinal changes like small and “dynamic” cysts not seen with OCT imaging and widespread areas of retinal edema related to swelling of retinal cells. Thus, subclinical microvascular changes in chronic diabetes can be detected in widespread regions of the retina with this technology. Even in minimal clinically visible retinopathy, it is now possible to visualize retinal vascular pathology with quantification of poor perfusion and ghost capillaries, blood vessel remodeling, and alteration of the blood–retinal barrier.

Although VEGF levels are increased in the vitreous of DME patients, there is a wide range of the VEGF levels, with some patients having very low levels and some very high levels of VEGF. Edward Feener (Joslin Diabetes Center, Boston, MA, USA) emphasized that the role of VEGF as a biomarker in DME is not established yet as vitreal VEGF concentration weakly correlates with macular thickness, or response to anti-VEGF therapy.^47^ The vitreous proteomic studies in DME patients have shown that VEGF level does not correlate with the majority of vitreous proteomic changes observed. Diabetic macular edema is associated with an array of cytokine changes (VEGF, monocyte chemoattractant protein [MCP-1], IL-6) in the aqueous; however, just as with VEGF, cytokine levels weakly correlate with macular thickness. Proteomic studies have identified over 2000 proteins in human vitreous and marked heterogeneity in proteomes among individual DR and DME patients. Total vitreous protein concentration is weakly associated with DME.^48^ Cross-sectional studies comparing proteomic changes in the vitreous in different stages of DR will likely provide further information on disease pathogenesis and may lead to new treatment and diagnostic modalities. Treatment strategies may be based on downregulating proteins involved in disease progression and/or upregulating proteins with a protective function. Further characterization of the vitreous in relation to other clinical features may result in biomarkers associated with disease progression, or the clinical response to therapies.

## Control of Systemic Factors

While many treatment modalities like laser, pharmacotherapies, and surgery are available for management of DR, the role of control of systemic factors has been found to be important as evidenced by large-scale epidemiologic studies. Emily Chew (National Eye Institute, Bethesda, MD, USA) discussed the results of the ACCORD Eye Study (Action to Control Cardiovascular Risk in Diabetes), a randomized controlled clinical trial that examined the benefits of intense blood glucose control, blood pressure, and serum lipid levels on retinopathy in type 2 diabetes patients.^49^ Intensive glycemia therapy (HbA1c level 6.4% in the intensive group versus 7.5% in the conventional group) significantly reduced the progression of DR (three steps in the ETDRS scale) by 35% over a 4-year span. However, the study did not demonstrate a significant effect of intensive versus standard blood pressure control on the progression of DR. The study found a beneficial effect of fenofibrate therapy on the progression of DR in those who were also receiving simvastatin. Chew also pointed out that tight glycemia control increased the mortality rate in these patients. The medical therapies had no effect on visual acuity, DME, and cataract surgery. Based on the results of the ACCORD Study, her clinical recommendations were that intensive glycemia control and the combination of fenofibrate and simvastatin are important in the treatment of DR, but the degree of intensive therapy will depend on the patient's health status and expectations. Intensive blood pressure control (below 140 mm Hg) may not be necessary because of the weak evidence of benefit from blood pressure control in the progression of DR.

In the same context, Robert N. Frank (Wayne State University, Detroit, MI, USA) further elaborated on lessons from clinical trials in DR in his special lecture titled “A History of Controlled Clinical Trials in the Treatment of Diabetic Retinopathy.” The lecture recognized his longstanding, enormous contributions to the field of DR in basic sciences as well as in clinical sciences. The first controlled trial in diabetes was the University Group Diabetes Program (UGDP) (1960–1970), which compared the effects of diet alone, tolbutamide, fixed-dose insulin, variable-dose insulin, and phenformin in reducing large vessel disease or death in recently diagnosed type 2 diabetes patients.^50^ The study resulted in a series of controversies as excessive deaths were reported in the phenformin group because of lactic acidosis and in the tolbutamide group because of cardiovascular events. Frank elegantly traced the history of the Airlie House Conference in 1968 that led to the large randomized trials like Diabetic Retinopathy Study (DRS) and Early Treatment Diabetic Retinopathy Study (ETDRS) showing the benefits of laser therapy.^51^ He pointed out that those landmark trials resulted in innovations like the ETDRS visual acuity chart and standardized grading scale of fundus photographs that were subsequently used in other trials. As he described results of important trials like DRS, ETDRS, DCCT, United Kingdom Prospective Diabetes Study (UKPDS), and ACCORD studies, he also emphasized the significance of establishing the DRCR (Diabetic Retinopathy Clinical Research) Network, the largest clinical network funded by NIH to explore clinical questions encountered in day-to-day practices in management of DR. Frank ended his lecture with unanswered questions for future studies: What percentage of clinically significant macular edema (CSME) patients are not successfully treated with anti-VEGF drugs, and how to treat them? How do we address genetic or epigenetic influences on the pathogenesis of DR? Is there a role of antioxidants in progression of DR?

## Laser and Surgery

The DRS concluded that panretinal laser photocoagulation (PRP) is effective in reducing vision loss by 50% in PDR patients. Daniel Palanker (Stanford University, Palo Alto, CA, USA) discussed that the PRP laser destroys a significant proportion of photoreceptors (30%), reducing retinal metabolic demand and decreasing production of angiogenic factors like VEGF. Histologic studies in animals showed that lighter lesions produced with shorter pulses avoid inner retinal damage and decrease scarring.^52^ Further, smaller and lighter lesions can even heal without scarring (with some RPE damage). The photosensitivity in the healing lesions is actually restored over time (1–4 weeks) due to redistribution of photoreceptors. Restorative photocoagulation may reduce night blindness and field loss seen after PRP laser, but the number of smaller lesions should be increased to maintain clinical efficacy.^53^ Focal/grid laser using small, light-intensity laser burns (50–100 μm in diameter) to microaneurysms and/or diffuse area of thickening in a grid pattern has been the gold standard treatment for clinically significant macular edema. Palanker explained how some of the laser-treated thermal complications such as subretinal fibrosis or enlargement of laser scars could be avoided by using “barely visible” (subvisible) and nondamaging therapy (NDT) (30% energy).^54^ The End Point Management (EpM) algorithm (100%) based on titration by barely visible burns provides reliable settings for subvisible and nondamaging treatment. The optimum energies for subvisible and NDT are 75% and 30%, respectively. The damage to RPE in small spots (subvisible clinically, but seen on fluorescein angiography [FA]) recovers within a week. Since pulse duration is below 15 ms, a larger number of exposures (400–600) can be rapidly applied using computer-guided pattern scanning laser. Clinical trials of laser nondamaging retinal laser therapy (NRT) (30% EpM) are in progress in many retinal diseases including DME, central serous retinopathy, age-related macular degeneration, macular telangiectasia, and branch retinal vein occlusion.

In advanced stages, many patients with proliferative DR end up in severe visual loss due to vitreous hemorrhage and tractional retinal detachment.^55^ In recent years, advancements in surgical instrumentations and novel techniques have significantly improved the outcomes in these patients. Gary Abrams (Wayne State University, Detroit, MI, USA) discussed the state-of-the-art instrumentations including small-gauge micro-incisional vitrectomy system along with wide-angle viewing with the BIOM system. He analyzed several dissection techniques like segmentation, delamination, en bloc dissection, or combination technique in diabetic vitrectomies. The smaller-gauge vitrectomy appears to have advantages as the membranes can be cut and removed with the vitrectomy cutter itself because its opening is closer to the tip of the cutter. The technique is faster, with less movement of instruments in and out of the eye, and often does not need sutures. High-speed vitrectomy cutters, dye-assisted vitrectomy, better illumination with the chandelier system, and preoperative use of anti-VEGF agents to control intraoperative bleeding have helped in achieving better outcome in diabetic vitrectomies. Abrams also presented results of the DRCR vitrectomy study in tractional DME, and both macular edema and vision improved after surgery with inner limiting membrane peeling. However, the indication of surgery in DME patients in absence of traction is controversial. He cautioned that eyes with severe ischemia, large intraretinal cysts, poor visual acuities, and more marked macular edema respond less well to vitrectomy. The role of vitrectomy in nontractional DME that persists after anti-VEGF treatment is still to be defined with future randomized clinical trials.

## Pharmacotherapies

Vascular endothelial growth factor (VEGF) is a potent vasopermeability factor that has been shown to be elevated in the vitreous of patients with DME. Several anti-VEGF drugs that target the VEGF molecule are available in the market: anti-VEGF aptamer pegaptanib (Macugen; OSI Pharmaceuticals, Farmingdale, NY, USA), the full-length anti-VEGF antibody, bevacizumab (Avastin; Genentech, San Francisco, CA, USA), the monoclonal anti-VEGF antibody fragment (Lucentis, Genentech), and the VEGF-Trap fusion molecule (Eylea; Regeneron, Tarrytown, NY, USA). Lloyd P. Aiello (Joslin Diabetes Center, Boston, MA, USA) discussed the paradigm shifts of the treatment of center-involving DME. The DRCR (Diabetic Retinopathy Clinical Research Network) Protocol I results showed that intravitreal ranibizumab with deferred laser was more effective in visual improvement through 5 years compared to ranibizumab with prompt laser. The 15-letter or more improvement in vision was seen in 27% to 38% of DME patients, and 50% of patients did not require laser over 5 years.^56^ Similar vision improvement was also seen in DME patients in the RIDE/RISE (ranibizumab versus sham injection) and VIVID/VISTA (aflibercept versus laser) studies. Out of these three drugs in DME patients, the use of intravitreal ranibizumab and aflibercept has been approved by the Food and Drug Administration (FDA), but intravitreal bevacizumab still remains an off-label drug. As bevacizumab is much cheaper than the two approved drugs, the DRCR conducted a head-to-head trial with these three drugs to compare their efficacy and safety in DME patients.^57^ When combined together, there was no difference in vision improvement among these three drugs. A subgroup analysis showed that in eyes with worse visual acuity (20/50 or worse), aflibercept was more effective in vision improvement, whereas in eyes with better visual acuity (equal to or greater than 20/40), there was no real difference among these three drugs. An interesting finding from these trials was that the progression of DR was lower, and the severity of DR (ETDRS severity scale) improved in the patients treated with anti-VEGF drugs. The DRCR Protocol I results indicated that there was almost a 50% decrease in worsening of DR in eyes with NPDR at baseline over 3 years with ranibizumab treatment and deferred laser.

As monthly anti-VEGF injections are a treatment burden, long-acting anti-VEGF drug delivery systems are being investigated. J. Jill Hopkins (Genentech, San Francisco, CA, USA) discussed the development of the Ranibizumab Port Delivery System (RPDS) in retinal diseases. This refillable, sustained delivery implant can be placed in the pars plana using standard vitreoretinal surgical techniques without scleral sutures. The implant can be refilled in the clinic using a custom needle that exchanges the implant contents with fresh ranibizumab. It has been studied in a phase I clinical study in wet AMD patients, and a phase II trial (LADDER, ClinicalTrials.gov NCT02510794) has been started. Sustained delivery potentially offers better long-term visual outcomes and disease modification as there is a more consistent pharmacokinetic profile with fewer peaks and troughs, and the treatment burden may be decreased.

**Figure 3:**
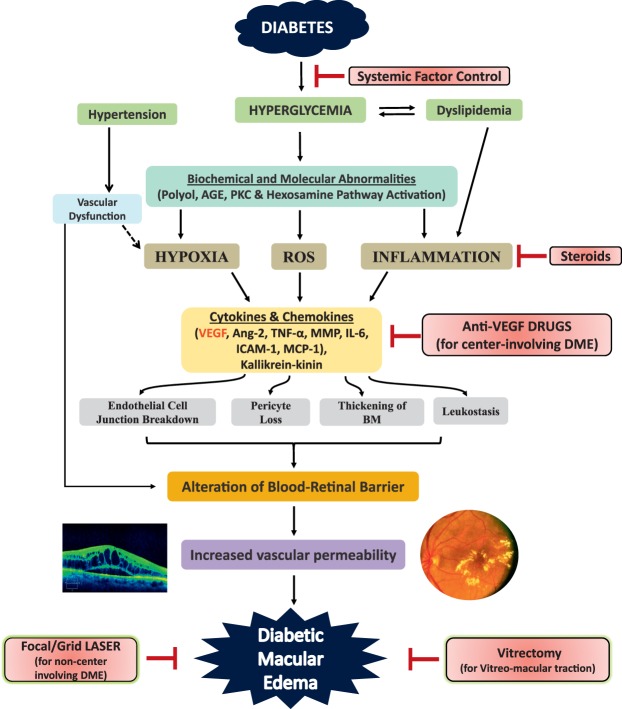
Pathophysiology of diabetic macular edema (DME) and different therapeutic strategies. Hyperglycemia in diabetes activates different biochemical pathways that lead to increased hypoxia, reactive oxygen species (ROS) formation, and inflammation with production of cytokines and chemokines. These mediators then cause endothelial cell junction breakdown and leukostasis, resulting in alteration of the blood–retinal barrier (BRB), increased retinal vascular permeability, and DME. Hyperglycemia also causes thickening of the basement membrane (BM) and pericyte dropout. Hypertension and hyperlipidemia, which coexist commonly in diabetic persons, further damage the already altered BRB. Currently, control of systemic factors, focal/grid laser (for noncenter-involving DME), anti-vascular endothelial growth factor (VEGF) therapies (for center-involving DME), steroids, and vitrectomy (in cases of vitreomacular traction) are the mainstays of management of DME. However, the current anti-VEGF therapies have limitations because they target only VEGF rather than other inflammatory molecules (e.g., angiopoietin [Ang]-2, matrix metalloproteinase [MMP], tumor necrosis factor [TNF]α, interleukin [IL]-1β, kallikrein–kinin) present in the retina in diabetic persons. Many future therapies target these other mediators. AGE, advanced glycation end products; ICAM, intercellular adhesion molecule; MCP, monocyte chemoattractant protein; PKC, protein kinase C. Reprinted with permission from Das A, McGuire PG, Rangasamy S. Diabetic Macular Edema: Pathophysiology and Novel Therapeutic Targets. *Ophthalmology*. 2015;122:1375–1394. Copyright © 2015 American Academy of Ophthalmology. Published by Elsevier, Inc. All rights reserved.

Although anti-VEGF treatment is an effective and safe therapy for center-involving DME, only 33% to 45% of patients on anti-VEGF drugs show three or more lines of visual improvement as shown in RIDE/RISE, VIVID/VISTA, and DRCR Protocol I trials. Arup Das (University of New Mexico, Albuquerque, NM, USA) stressed that DME is a heterogeneous disease and that multiple cytokines and chemokines play an important role in its pathogenesis.^58^ Steroids have been found to be effective in vision improvement and slowing down the progression of DR, although there are potential side effects of cataract formation and increase in IOP. Das discussed the novel therapeutic targets beyond VEGF and focused on three important pathways (Fig. 3). First, the level of angiopoietin-2, a strong vasopermeability factor, has been found to be elevated in conditions with vascular leakage (sepsis, disseminated intravascular coagulation, postcardiac arrest syndrome, acute kidney injury). In diabetic animals, angiopietin-2 is upregulated in retinal tissues, and similarly, hyperglycemia induces the expression of Ang-2 in isolated retinal endothelial cells.^59^ Thus, Ang-2 appears to play an important role in increased vasopermeability in DR, and thus appears to be an attractive target in addition to VEGF. Three clinical trials targeting the Ang-2/Tie-2 pathway are currently ongoing in DME patients. The second important molecule in the inflammatory cascade in DR is the chemokine ligand (CCL2), also known as monocyte chemoattractant protein (MCP-1). The CCL2 is upregulated significantly in retinas of diabetic animals, and it results in increased monocyte trafficking as well as activation of microglia.^60^ The CCL2 has been targeted in clinical trials in patients with atherosclerosis, diabetic nephropathy, chronic kidney disease, and diabetes itself. At least in CCL2 knockout mice made diabetic, there is a significant reduction of retinal vascular permeability and less infiltration of monocytes in retinas. The third important pathway that could be a therapeutic target is the kallikrein–kinin pathway. The vitreous proteomics study has shown that there is increased plasma prekallikrein and kallikrein level in the vitreous of DME patients.^47^ A phase I clinical trial using intravitreal injection of plasma kallikrein inhibitor, KVD001 (PKal) (Kalvista, Cambridge, MA, USA) in DME patients has been completed, and a phase II trial is being planned.

Peter Campochairo (Johns Hopkins University, Baltimore, MD, USA) further discussed the role of a small-molecule Tie-2 activator (AKB-9778) (Aerpio, Cincinnati, OH, USA) that has been shown to suppress retinal neovascularization and leakage in mouse models. The Tie-2 pathway is also regulated by vascular endothelial protein tyrosine phosphatase (VE-PTP) that is physically associated with Tie-2. Levels of both Ang-2 and VEPTP are increased by hypoxia, resulting in VEGF-mediated ocular neovascularization and vascular leakage. The AKB-9778 drug targets the VE-PTP enzyme activating the Tie-2 pathway to stabilize retinal vessels.^61^ This drug given by subcutaneous injection has an excellent therapeutic index and had additive effects when given in conjunction with anti-VEGF drugs. A phase I study demonstrated safety and biologic activity of the AKB-9778 drug, and a phase II trial (TIME-2) was started (this trial recently finished, showed significant reduction of macular edema when used in combination with intravitreal ranibizumab).^62^ Thus, inhibition of VE-PTP with AKB-9778 restores Tie-2 activation and seems to be an appealing strategy in DR.

## Future Directions

### Telemedicine

Only ∼60% of the US diabetic population receives the recommended annual eye examinations, and this patient noncompliance is due, in part, to the absence of visual symptoms. Current treatments combined with telemedicine may reduce the incidence of diabetes-related blindness by as much as 95%. Paolo Silva (Joslin Diabetes Center, Boston, MA, USA) explained that telemedicine can help in diagnosing DR in early stages. Telemedicine has three key advantages: The images can be remotely acquired; there is centralized evaluation of images; and communication of findings to a remote site is easy. Even in a tertiary academic setting, an ocular diabetes telemedicine program can enhance access to eye care for patients with early retinopathy. In these individuals with early retinal disease, patient education, routine eye care, and medical management should be effective in preserving vision. Silva described his study comparing the efficiency of nonmydriatic ultrawide-field retinal imaging (UWFI) and nonmydriatic fundus photography (NMFP) in a DR ocular telehealth program. The study concluded that in a standardized DR ocular telehealth program, nonmydriatic UWFI reduced the ungradable rate by 71% (to <3%) and reduced image evaluation time by 28%. Diabetic retinopathy was identified 17% more frequently after UWFI, and DR peripheral lesions suggested a more severe DR level in 9%. These data suggest that UWFI may improve efficiency of ocular telehealth programs evaluating DR and DME.^63^ Silva also emphasized that telemedicine programs can readily be integrated into both tertiary and primary care settings, providing improved access and increased identification of eye diseases. With the growing adoption of telemedicine programs, methods of quality control and quality assurance need to implemented to maximize outcomes. With immediate image evaluation at the point of care, less than 0.01% of patients with referable DR would be missed while the reading center image grading burden would be reduced by approximately 60% and patient feedback expedited.

### Stem Cell Therapies

Diabetes is associated with endothelial dysfunction and decreased repair by vascular progenitors. Studies by Parsons-Wingerter et al.^64^ suggest that individuals with diabetes exhibit retinal vascular repair. Distinct vascular reparative populations are derived from the bone marrow (CD34^+^ cells) and the vessel wall (endothelial colony-forming cells [ECFCs]).^65^ CD34^+^ cells serve to provide paracrine support to the injured vasculature and are hematopoietic in origin as indicated by CD45^+^ surface expression. In contrast, ECFCs derived from vessels directly have high clonal replating capacity and expansion capacity in vitro. Individuals with diabetes and vascular complications have reduced numbers of both CD34^+^ cells and ECFCs. CD34^+^ cell number is low in the circulation of NDPR patients,^66,67^ and these cells demonstrate reduced endothelial nitric oxide synthase (eNOS) expression and increased ROS. This reduction in “bioavailable nitric oxide (NO)” leads to marked dysfunction in these cells, including reduced homing to areas of injury and ischemia.^68,69^ These cells also show reduced colony formation and inability to differentiate into endothelial cells.^70^ In addition to functional changes, these cells are unable to exit the bone marrow to enter the circulation due to denervation of the bone marrow and loss of circadian rhythmicity of their release.^71^ Maria Grant (Indiana University, Indianapolis, IN, USA) emphasized that autonomic centers in the brain regulate the release of vascular progenitors (CD34^+^ cells) from the bone marrow and also the release of inflammatory cells such as monocytes. In diabetes, neuroinflammation of the autonomic centers results in early sympathetic hyperactivity with too much release from the bone marrow and in late stages with neurodegeneration and too little release. Strategies to regulate not only retinal inflammation but also central nervous system inflammation may represent novel approaches to treating DR.^72–74^

### Genomic Approach

Diabetes is a multifactorial disease, and genetic factors play a significant role in the pathogenesis of DR as shown in some sporadic studies. Anand Swaroop (National Eye Institute, Bethesda, MD, USA) stressed that integrating multilevel data sets such as those in genome-wide association studies (GWAS), exome sequencing, genome sequencing, and targeted sequencing can advance the field. The concept of network biology in medicine and computational biology is infiltrating all aspects of medicine. Next-generation sequencing (NGS) technology, together with novel methods of pattern recognition and network analyses, has revolutionized the way fundamental biological mechanisms and cellular pathways are considered. Gene regulatory networks govern differentiation of retinal photoreceptors and modulate adaptive response during aging and diseases such as DR, and even therapies can be based on network biology. Basic strategies for network construction and analyses can be brought to any tissue or cell type. Both precise and consistent guidelines are needed for generation of genome, transcriptome, and epigenome data to facilitate comparative analysis and integration of multidimensional data sets, and for constructing networks underlying complex biological processes. Network-based biology will provide a foundation for deciphering disease mechanisms, including DR, and facilitate the discovery of novel drug targets for retinal diseases.^75^

### Drug Delivery

Ashwath Jayagopal (Roche Laboratory, Basel, Switzerland) covered the topic of emerging platforms for therapy of DR and the use of sustained-release/long-acting therapeutics. These agents provide benefit because of their reduced dosing frequency, their long-term bioavailability, and their enhanced drug stability in vivo. An example is the encapsulation of bevacizumab into slow-release polymeric microparticles that are solvent-free and amenable to industrial scale-up.^76^ Sustained-release technologies include hydrogels and drugs that are “encapsulated” in hydrogel networks.^77^ Jayagopal provided the example of K5-NP, a long-acting therapeutic that can inhibit VEGF and ICAM in a STZ model of DR.^78,79^ He also discussed the concept of cellular/molecular targeting of drugs to reduce off-target effects and dosage so as to confine therapy within target cells and presented the concept of “disease-specific drug activation.” He demonstrated macrophage /microglia targeting in the retina with CD105-targeted nanocarriers loaded with VEGFR2 siRNA that reduced the number of neovascular lesions in the oxygen-induced retinopathy (OIR) model.^80^

Targeted nanocarriers address key barriers toward clinical translation of nucleic acid therapies in that they provide specific, intracellular delivery of siRNA within dysfunctional endothelial cells in preretinal or subretinal neovascularization. They are biocompatible and are suited for multiple administration routes. The modular design allows for swapping targeting antibody, nucleic acid cargo, and anti–mir-RNA). This approach minimizes dosage and allows for combinative therapies.

Jayagopal presented an example of imaging of hypoxic tissues using the HYPOX Imaging Agent in vitro.^81,82^ He also described new technology that uses hypoxia-dependent fluorescence enhancement in vitro and in vivo as demonstrated by homing of intravenously injected small molecule exclusively within hypoxic retina in mouse model of OIR. The potential of hypoxia-targeted therapy includes (1) targeted delivery of drugs to diseased cells prior to onset of advanced disease; (2) drug/dye combinations for image-guided therapy (e.g., prophylactic laser); and (3) chemically coupled therapeutic modalities to hypoxia-targeted moieties to enhance bioavailability of drugs in hypoxic retina and (4) applicable to other retinal vascular diseases. Jayagopal also described other emerging therapies for DR including ROS-responsive polymers for drug depot release,^83^ catalytic antioxidant nanoparticles, matrix metalloprotease (MMP)-activated binding of drug delivery vehicles to diseased tissue,^84,85^ and therapies to leukocyte subpopulations using ligand-targeted nanoparticles (sugars, antibodies, peptides, aptamers)^86^ and pericyte-targeted therapies. Questions raised from this presentation include the following. How soon will we experience the impact of nanomedicine and nanotechnology in the form of paradigm-shifting clinical therapeutic interventions? What is the regulatory agencies' current view of application in the clinic? Does approval of nanomedicine in ophthalmology require “a new set of rules” not applicable to typical small molecules and biologics, for example, FDA review?

## More Questions

The session concluded with a series of questions for future discussion. The questions centered around the role of neurodegeneration, permeability, and inflammation. The following questions were discussed:

- Is the instigating event in the development of DR damage to the neural system or vascular system?
- Is there usefulness to hypoxia models or models of ischemia/reperfusion? Can we use these models to examine accelerated changes instead of the “truly” diabetic models? Is there a need to also validate these findings in hyperglycemic animals? What is best animal model of DME in the laboratory?
- What is the role of epigenetics in the pathogenesis of DR? Can mitochondrial epigenetics be targeted as one of the therapeutic targets? What is the role of mechanical control of endothelial cell activation in DR?
- Assuming that DR is the outcome of chronic inflammation, what does initiate the inflammation? What cell types are important for induction and maintenance of inflammation—tissue versus systemic? What role does disturbance of crosstalk among retinal cells play in inflammation induction? How does diabetes seemingly cause different inflammatory events (tissue versus systemic), and how do these distinct inflammatory events promote DR?
- Photoreceptors are an important source of diabetes-induced ROS and contribute to the proinflammatory retinal environment. How can we protect them better?
- Have we identified two cohorts of DME patients—one driven by VEGF and one by the kallikrein–kinin system?
- Now that we have newer ways of visualizing clinical pathology, is it time to reassess the endpoints used in clinical trials?
- Can we restore or regenerate defective vessels to promote blood flow into poorly perfused areas to abrogate the need for anti-VEGF therapy?
- While genomic and genetic analytical approaches have greatly improved our understanding of disease pathogenesis, how many of these approaches can be successfully integrated within the routine clinical diagnostic setting? Can all patients in every clinic get their DNA sequenced for personalized health care, in a cost-effective way, given the current technological landscape?
- Clinically, how do we move to personalized medicine to identify the best therapeutic approach to control or prevent DME?

In recent years, we have seen a sudden momentum in exploring novel pharmacotherapies, drug delivery systems, sustained-release medications, stem cell therapy, high-resolution imaging systems, subthreshold laser, and microincisional surgeries to treat DR for vision improvement. With the steep increase in the incidence of diabetes globally, there is an increased need for networking and collaborative efforts among clinicians, basic scientists, pharmaceutical industries, and regulatory agencies. With an estimate of more than half a billion diabetics by 2040, we need approximately 2000 eye examinations per minute to meet the goal of once-a-year eye examination. The question remains: Are we ready to fight this global epidemic? The ARVO Conference brought together scientists from all fields who shared their thoughts and were engaged in an intimate discussion to explore novel ideas and meet the challenges of the current therapies. With a limited number of ophthalmologists that is not increasing at the same pace as the diabetes epidemic, we are far behind in providing the basic needs for a minimum of one eye examination for all the diabetics in the world. Telemedicine with its expansion into the automated reading system is facing its challenges to meet this big need for screening diabetic patients in remote rural and urban populations. More focused group meetings like this conference to initiate brainstorming for ideas to meet the challenges are needed, and the new frontier of research lies in this multidisciplinary approach to develop innovative technologies to fight the global epidemic of diabetes that has a multitude of devastating complications in many target organs.
